# Editorial: Patient and public involvement in disability and rehabilitation research

**DOI:** 10.3389/fresc.2023.1307386

**Published:** 2023-11-14

**Authors:** Thilo Kroll, Reuben Escorpizo

**Affiliations:** ^1^ Midwifery and Health Systems, School of Nursing, University College Dublin, Dublin, Ireland; ^2^ Department of Rehabilitation and Movement Science, The University of Vermont, Burlington, VT, United States

**Keywords:** disabilities, rehabilitation research and development, patient and public involvement, patient and public engagement, human rights

**Editorial on the Research Topic** Patient and public involvement in disability and rehabilitation research

Patient and Public Involvement (PPI) has gained momentum over the past two decades in health service planning and health-related research. The collection for this Research Topic focuses on how stakeholders can be meaningfully involved in disability and rehabilitation research. It contains eight articles consisting of one study protocol, four perspective articles, and three original research papers. Each paper provides a unique focus ranging from the value- and rights-based foundations of PPI to practice examples on how PPI can be purposefully and effectively realised.

The paper by Browne and Dorris examines PPI from a human rights-based perspective in line with the UN Convention on the Rights of Persons with Disabilities (UN CRPD). They argue that people with disabilities have a right in key decisions about research, and that the research community has a legal obligation to ensure that research processes are inclusive. As most UN member states have signed and ratified the UN CRPD, efforts need to be strengthened to ensure accessibility (i.e., Article 9) and equality of opportunities (i.e., Article 3e) for people with disabilities.

Kerr et al. discuss how PPI can be meaningfully and effectively embedded in draft standards that guide research of the Northern Ireland Cerebral Palsy Research Registry (NICPRR). While the standards have proven to be helpful in advancing PPI in the NICPRR research, challenges remain in terms of sustainable funding and training support for PPI representation in research planning and conduct.

Manikandan et al. demonstrate in their article how value-based, mutually beneficial involvement of people with cerebral palsy can be realised in the context of doctoral research. They characterise the experiences through the life cycle of a project that took place during the COVID-19 pandemic, both from the perspective of the doctoral student and individuals with cerebral palsy. The paper shows how critical the management of expectations from different stakeholders is.

Central to PPI is the quality of relationships between academic and non-academic partners in the team. Critically, people with disabilities bring a unique understanding to the team and can share issues that may remain otherwise hidden to the academic researcher. Herrman et al. report on a community-based peer navigator model that seeks to improve health care access for Medicaid beneficiaries with disabilities in the United States. Peer navigators formed deep, supporting relationships with peers and the study uncovered multiple social challenges, including poverty, social isolation, and racial- and disability- related discrimination. As they were also closely working with the academic team, changes could be made to the peer program to reflect a greater emphasis on the social determinants of health.

Creative approaches including art, photography and music have a long tradition in participatory research. Rose et al. have engaged people with Parkinson's Disease and caregivers in participatory workshops with researchers to identify preferences and benefits of music for mood and movement. The workshops served to reduce the power imbalance between academics and non-academics and led to an enhanced understanding of each other's priorities.

Collaborative or participatory design can be a powerful approach in PPI as researchers, professionals and people with disabilities see quick and tangible benefits of their work together. Hall et al. describe in their article how people who experienced a stroke can share their life stories and experiences with rehabilitation to develop digital education tools for health care professionals. The individual life stories generate powerful opportunities for empathy and bespoke responses in rehabilitation education and practice.

Co-Design as a powerful involvement approach can work at any age and with any population. The article by Fortune et al. shows how young people with cerebral palsy were involved to co-design resources that could support transition from paediatric to adult health services. The team followed a structured but iterative design thinking approach that also included health care professionals.

There is a fundamental need to build capacity for PPI among students and early career researchers in rehabilitation sciences. The work by Horgan et al. provides a roadmap and protocol on how PPI can be assessed and evaluated in the context of a structured PhD program for stroke care.

The papers in this Research Topic demonstrate that PPI in rehabilitation research is not only feasible but that it can also enhance the quality of research. It can unveil stakeholder-centric determinants of health that otherwise would be hidden to the researcher, and it can enhance acceptance and usability of products and services if people with disabilities are involved from the start. People empowerment is a natural consequence of PPI. Perhaps, most fundamentally, involvement is not only an ethos but a human right and legal obligation. It also must be stated that PPI have yet to reach maturity in rehabilitation sciences despite its visibility in many fields of health research. Hence, we must continue to promote, advocate for, and integrate PPI in rehabilitation research.

